# The Synthetic Amphipathic Peptidomimetic LTX109 Is a Potent Fungicide That Disturbs Plasma Membrane Integrity in a Sphingolipid Dependent Manner

**DOI:** 10.1371/journal.pone.0069483

**Published:** 2013-07-12

**Authors:** Rasmus Bojsen, Rasmus Torbensen, Camilla Eggert Larsen, Anders Folkesson, Birgitte Regenberg

**Affiliations:** 1 Department of Systems Biology, Technical University of Denmark, Kgs. Lyngby, Denmark; 2 Department of Biology, University of Copenhagen, Copenhagen, Denmark; 3 Section for Bacteriology, Pathology and Parasitology, National Veterinary Institute, Frederiksberg C, Denmark; Simon Fraser University, Canada

## Abstract

The peptidomimetic LTX109 (arginine-tertbutyl tryptophan-arginine-phenylethan) was previously shown to have antibacterial properties. Here, we investigated the activity of this novel antimicrobial peptidomimetic on the yeast *Saccharomyces cerevisiae*. We found that LTX109 was an efficient fungicide that killed all viable cells in an exponentially growing population as well as a large proportion of cells in biofilm formed on an abiotic surface. LTX109 had similar killing kinetics to the membrane-permeabilizing fungicide amphotericin B, which led us to investigate the ability of LTX109 to disrupt plasma membrane integrity. *S. cerevisiae* cells exposed to a high concentration of LTX109 showed rapid release of potassium and amino acids, suggesting that LTX109 acted by destabilizing the plasma membrane. This was supported by the finding that cells were permeable to the fluorescent nucleic acid stain SYTOX Green after a few minutes of LTX109 treatment. We screened a haploid *S. cerevisiae* gene deletion library for mutants resistant to LTX109 to uncover potential molecular targets. Eight genes conferred LTX109 resistance when deleted and six were involved in the sphingolipid biosynthetic pathway (*SUR1*, *SUR2, SKN1*, *IPT1*, *FEN1* and *ORM2*). The involvement of all of these genes in the biosynthetic pathway for the fungal-specific lipids mannosylinositol phosphorylceramide (MIPC) and mannosyl di-(inositol phosphoryl) ceramide (M(IP)_2_C) suggested that these lipids were essential for LTX109 sensitivity. Our observations are consistent with a model in which LTX109 kills *S. cerevisiae* by nonspecific destabilization of the plasma membrane through direct or indirect interaction with the sphingolipids.

## Introduction

Infections caused by pathogenic yeast such as *Candida spp*. affect a large number of immunosuppressed patients and are an increasing medical problem [1], [2]. Fungal infections are currently treated with one of four major classes of antifungals. Azoles target ergosterol synthesis [3], polyenes bind to ergosterol in the cell membrane and form pores [4], [5], echinocandins inhibit cell wall synthesis [6], and 5-fluorocytosine interferes with protein and DNA synthesis [7].

Decreased susceptibility to the most frequently used antifungal, fluconazole, has recently been reported, and the number of nonsusceptible *C. glabrata* isolates from humans is increasing [8], [9]. Resistance towards 5-fluorocystosine is also rapidly developing [10]. Polyenes can be toxic [11] and echinocandins have a narrow spectrum of activity [12]. An additional complication in the treatment of nosocomial fungal infections is the frequent formation by fungi of sessile communities called biofilms in association with medical implants [13]. Limited nutrient access leads to slow-growing, antibiotic tolerant cells in biofilms that can serve as a reservoir for infection [14], [15]. Most systemic antifungals are fungistatic against yeasts, so they are primarily effective against actively growing cells and have poor activity against cells in biofilms.

The limited number of antifungal classes and drugs with fungicidal properties raises the need for novel drugs with activity against slow-growing and biofilm-forming pathogenic fungi [16], [17]. Antimicrobial peptides (AMPs) and modified forms of AMPs offer an attractive alternative to conventional antifungal drugs. AMPs are cationic and amphipathic peptides of 12–50 amino acids that are produced by species in almost every kingdom and phylum of life [18]. The amphipathic structure of AMPs suggests that they might have targets that are different from conventional antifungals [19], [20]. The high degradation rate of many natural AMPs can be circumvented by backbone and side chain alterations that create structural analogs that mimics natural peptides [21]. A number of synthesized peptidomimetics have *in vitro* antifungal activity, making these compounds attractive candidates for novel antifungal drugs [22]–[24].

We tested the antifungal activity of the short, antibacterial peptidomimetic LTX109 (arginine-tertbutyl tryptophan-arginine-phenylethan). LTX109 is based on an Arg-Trp-Arg sequence found in the AMP bovine lactoferricin and was originally developed as an antibacterial [25]–[27].

We used killing kinetics to describe the antimicrobial effect of LTX109 and investigated its mode of action by measuring transport of H^+^, K^+^, amino acids and a fluorescent dye across the cell membrane. To uncover potential molecular targets that would explain the fungicidal activity of LTX109, we screened a *Saccharomyces cerevisiae* gene deletion library for mutants resistant to LTX109. Most mutations that led to LTX109 resistance were in genes involved in the synthesis of the sphingolipids mannosylinositol phosphorylceramide, MIPC, and mannosyl di-(inositol phosphoryl) ceramide, M(IP)_2_C. These results indicate that M(IP)_2_C and/or MIPC in the plasma membrane are essential for the action of LTX109.

## Materials and Methods

### Strains, growth media and antifungal drugs

The S288c *S. cerevisiae* strain M3750 (*MAT* **a** *ura3-52*) [28] was used as the reference strain in all experiments unless otherwise indicated, while the barcode-tagged deletion-mutant library was from Johnston and coworkers [29]. Σ1278*b* (10560-2B; *MAT* **a** *ura3-52 leu2:hisG his3:hisG*) was used for biofilm susceptibility experiments [30]. Complex YPD medium [31] was used in all experiments except for amino acid release and biofilm where cells were grown in synthetic complete medium [31]. LTX109 (Lytixar; LytixBiopharmaAS, Oslo, Norway) and amphotericin B (Sigma) were dissolved in water and stock solutions were kept at −20°C.

### Broth microdilution minimal inhibitory concentrations

Minimal inhibitory concentration (MIC) values were measured under static conditions in polystyrene microtiter plates. Two-fold dilution series of antifungal drug were prepared in fresh YPD medium and distributed to microtiter-plate wells. Overnight cultures of wild type (WT) S288c were diluted and added to antifungal-containing wells to a final concentration of 2×10^5^ cells/ml. Growth inhibition was recorded with absorbance at 600 nm after 24 hours at 30°C. The lowest drug concentration resulting in 90% growth inhibition was the MIC. MIC values of LTX109 were determined three times with triplicate measurements, while MIC values of amphotericin B was determined once with triplicate measurements.

### Killing kinetics

Overnight cultures of *S. cerevisiae* were diluted in fresh, preheated YPD to 4×10^5^ cells/ml and incubated at 30°C with aeration. Exponential growth phase cells were challenged with LTX109 or amphotericin B at concentrations that were five times the MIC. Control samples were treated with water to ensure that cells applied in the time-kill experiment were in exponential growth phase. Samples were taken at the indicated time points, diluted 10-fold, and plated on YPD agar to determine colony forming units (CFUs). The time-kill experiment was conducted in triplicates.

### Acidification assay

Glucose-induced acidification was measured as previously described [32] with modifications. Exponentially growing *S. cerevisiae* cells were washed and resuspended in sterile water to a final concentration of 10^8^ cells/ml. Cells were subsequently incubated with LTX109 (100 µg/ml) or water (control) for 10 minutes before the assay was initiated by addition of glucose to a final concentration of 2% (w/v). The assay was conducted in triplicate at room temperature with continuous magnetic stirring. The assay was stopped by sampling at indicated time points, followed by immediate centrifugation (2000×*g* for two minutes). pH of the resulting supernatants was measured and changes in extracellular H^+^ concentration were calculated by applying the obtained values to the equation pH = −log [H^+^].

### Potassium release

Exponentially growing *S. cerevisiae* were harvested and resuspended in sterile water as described above. The potassium release assay was initiated by addition of LTX109 to a final concentration of 10 times the MIC. Samples treated with water instead of LTX109 served as control. The assay was stopped by centrifugation of samples (13,000×*g* for 1 min) at indicated time points. Supernatants were transferred to sterile microtubes for spectrometric analysis. Potassium concentrations were measured with a FLM3 flame photometer (Radiometer). A standard concentration curve was generated from diluted S3336 urine flame standard (Radiometer). For spectrometric analysis, 5 µl of sample was added to 1000 µl of S3336 lithium solution (Radiometer). Experiments were carried out in triplicates at room temperature.

### SYTOX Green uptake

SYTOX Green uptake was measured as previously described [33] with modifications. Exponentially growing *S. cerevisiae* cells were centrifuged, washed and suspended in 5 µM SYTOX Green (Life Technologies) to a final concentration of 10^8^ cells/ml. LTX109 or water (control) was added to cell suspensions and SYTOX Green uptake was recorded microscopically after 4, 8, 16, 32, 64 and 128 minutes. Fluorescence was recorded with a Nikon Eclipse (Tokyo, Japan) fluorescence microscope equipped with a F36–525 EGFP HC-filter set (AHF Analysentechnik). Experiments were carried out at room temperature. SYTOX green uptake upon LTX109 treatment was observed in three independent experiments.

### Amino acid release

Exponentially growing *S. cerevisiae* were harvested and suspended in sterile water or water with 10 times the MIC of LTX109 to a final concentration of 2×10^6^ cells/ml. Loss of free amino acids from cells was recorded at room temperature after 16 minutes LTX109 exposure by instant centrifugation and subsequent HPLC of the cell free supernatant. Amino acids were detected and quantified by reverse-phase HPLC using an LKB-Alpha Plus amino-acid analyzer and a mixture of L-α-amino acids, 1 nmol each, as standards. The experiment was repeated three times.

### Identification of LTX109-resistant mutants

Haploid knockout mutants of approximately 4000 nonessential genes in the S288c deletion mutant library [29] were pooled. About 10^6^ cells from the mutant pool were transferred to YPD agar containing 10 times the MIC of LTX109. After 72 hours at 30°C, 17 colonies were picked from the LTX109 plates. LTX109-resistant clones were identified by PCR amplification and Sanger sequencing of the unique barcode tag of each mutant. PCR templates were DNA from clonal isolates of LTX109-resistant mutants. Primers were 5′-GATGTCCACGAGGTCTCT and 5′-CTGCAGCGAGGAGCCGTAAT. Gene deletions were identified using barcode sequences in the Stanford SGD deletion database.

### Spot test

Exponentially growing *S. cerevisiae* cells were diluted to 10^7^ cells/ml and 6 µl of a 10-fold dilution series was spotted on YPD agar and YPD agar containing 10 times the LTX109 MIC. Plates were incubated for 24 hours at 30°C and growth results were recorded.

### Biofilm susceptibility

*S. cerevisiae* (Σ1278*b*) cells were grown in Lab-Tek™ Chamber Slide™ System; Permanox® (NUNC, Denmark) [34], [35] in 1 ml synthetic complete medium. Cells were initially allowed to form biofilm for 12 hours before LTX109 was added for 5 hour in a concentration of 0 or 70 µg/ml. The biofilm was subsequently stained 15 minutes with Syto 9 (Invitrogen, Irvine, CA) for life cell staining and propidium iodine for dead cell staining before confocal laser scanning microscopy (CLSM). Imaging was carried out using a 63x/0.95NA water immersion lens. CLSM was performed with a Zeiss LSM510 microscope. Staining of biofilm treated with LTX109 was repeated in four independent experiments.

## Results

### Fungicidal properties of LTX109

**Figure 1:**
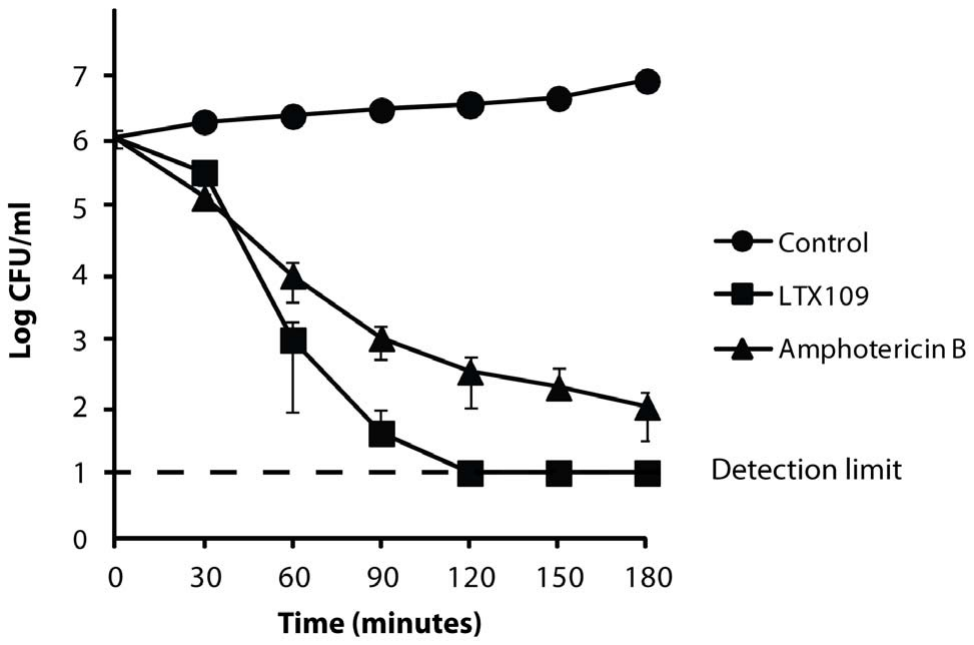
Fungicidal properties of LTX109 and amphotericin B. Time-kill kinetics of exponentially growing yeast cells exposed to water (circles) or five times the MIC of LTX109 (40 µg/ml) (squares) or amphotericin B (10 µg/ml) (triangles). Viability was examined every half hour as CFUs. Each data point is the average of three individual measurements ± standard deviation.

We tested the antifungal properties of the peptidomimetic LTX109 on the yeast *S. cerevisiae* using microdilution. LTX109 had antifungal activity against *S. cerevisiae* at 2×10^5^ cells/ml with a MIC value of 8 µg/ml compared to 2 µg/ml amphotericin B. Assay to determine the killing kinetics of LTX109 against *S. cerevisiae* revealed rapid and efficient fungicidal properties resulting in a 3-log reduction in viable cells within one hour, while amphotericin B required 90 minutes to achieve a similar fungicidal effect when using drug concentrations in multiples of MIC (Fig. 1). Additionally, LTX109 reduced the yeast population to the detection limit within only 2 hours, an effect that was not achieved by amphotericin B in the first 3 hours of exposure.

### Exposure to LTX109 disrupts plasma membrane integrity

The speed with which LTX109 killed *S. cerevisiae* suggested that the compound was acting directly on the plasma membrane. To investigate the effect of LTX109 on plasma membrane integrity, we measured H^+^ efflux, ion loss, loss of amino acids and uptake of the fluorophore SYTOX Green across the plasma membrane.

**Figure 2:**
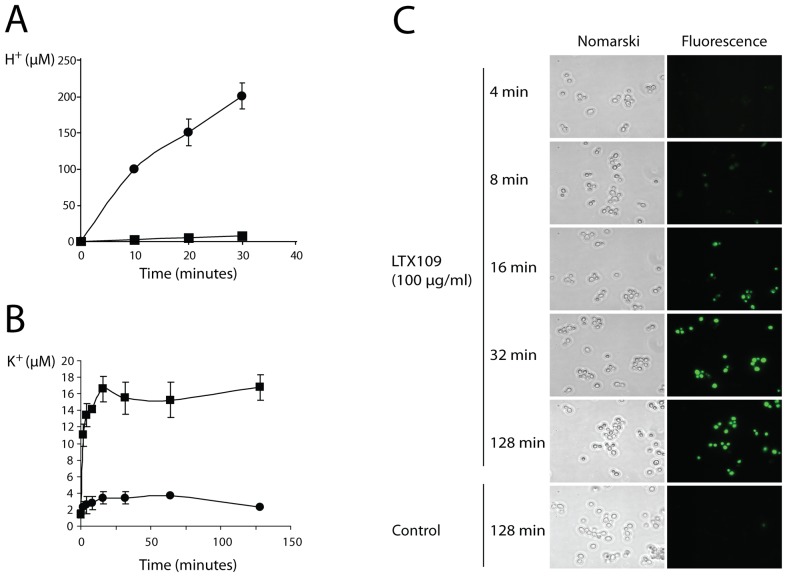
Transport of H^+^, K^+^ and a fluorescent dye by cells treated with LTX109. (A) Glucose-induced acidification of medium by yeast cells. Exponentially growing *S. cerevisiae* was washed and suspended in sterile water and exposed to 100 µg/ml LTX109 (squares) or water (circles) before glucose addition at time zero. Medium pH was measured and H^+^ concentration calculated from pH = −log [H^+^]. Each data point is the average of three individual measurements with standard deviations as error bars. (B) Potassium release from yeast cells. Exponentially growing yeast cells were washed, resuspended in water, and challenged with 100 µg/ml LTX109 (squares) or water (circles) at time zero. Potassium release was measured using flame atomic absorption spectrometry in binary increasing intervals. Each data point is the average of three individual measurements ± standard deviation. (C) Nomarski (left) and fluorescent (right) microscopy of SYTOX Green-stained yeast cells. Exponential growing cells were exposed to 100 µg/ml LTX109 and SYTOX Green uptake was monitored. Cells treated with SYTOX Green and 0 µg/ml LTX109 served as control. SYTOX green uptake upon LTX109 treatment was observed in three independent experiments.

Yeast cells treated with glucose acidify their surroundings primarily by active transport of H^+^ by the plasma membrane H^+^-ATPase [36]. We found that glucose-induced acidification was completely absent when cells were treated with LTX109 for 10 minutes before glucose addition (Fig. 2A). These results suggested that LTX109 decoupled the plasma membrane potential directly or indirectly by inhibition of e.g. ATP synthesis.

We next tested the loss of potassium from cells treated with LTX109. Potassium release occurred immediately and increased during the first 16 minutes of exposure to LTX109, reaching a steady state that was more than four times higher than the maximum of the untreated control (Fig. 2B). Much of the K^+^ that was lost was detected within the first two minutes of challenge with a high LTX109 concentration. These results suggested that LTX109 acted by direct interaction with and disturbance of the plasma membrane rather than through indirect inhibition of metabolism or another intracellular pathway.

**Figure 3:**
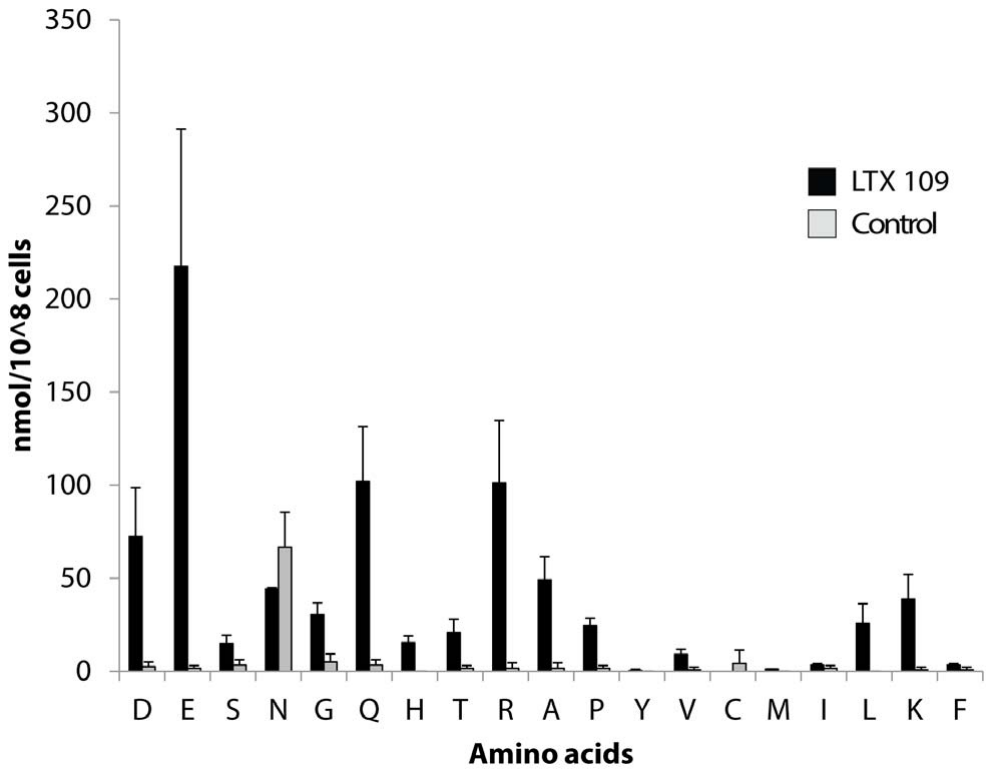
Efflux of amino acids from cells treated with LTX109. Exponentially growing yeast cells were washed, resuspended in water, and challenged with 70 µg/ml LTX109 (black bars) or water (grey bars) for 16 minutes. Amino acids (one letter code) in the extracellular medium were subsequently measured by HPLC. Each data point is the average of three individual measurements ± standard deviation.

To investigate if LTX109 treatment also led to loss of other small molecules, cells were treated with LTX109 for 16 minutes and free amino acids measured in the extract. Yeast cells treated with LTX109 lost substantial amounts of at least 14 different amino acids whereas cells treated with water only leaked aspartate (Fig. 3). The loss corresponds well to the pool of intracellular amino acids found in other experiments [37], suggesting that most if not all free amino acids are depleted from cells treated with LTX109.

We finally investigated if the membrane potential decoupling was a consequence of plasma membrane destabilization by monitoring the uptake of the 600-Dalton nucleic acid stain SYTOX Green. SYTOX is an inorganic compound that fluoresces upon DNA binding and SYTOX Green can only enter a cell and fluoresce if the plasma membrane is compromised [38]. We found that untreated cells were impermeable to SYTOX Green, while cells treated with LTX109 became permeable. The dye was visible in the nucleus of LTX109-treated cells after only eight minutes (Fig. 2C) and fluorescence increased with LTX109 exposure time.

### Defects in sphingolipid synthesis lead to LTX109 resistance

**Figure 4:**
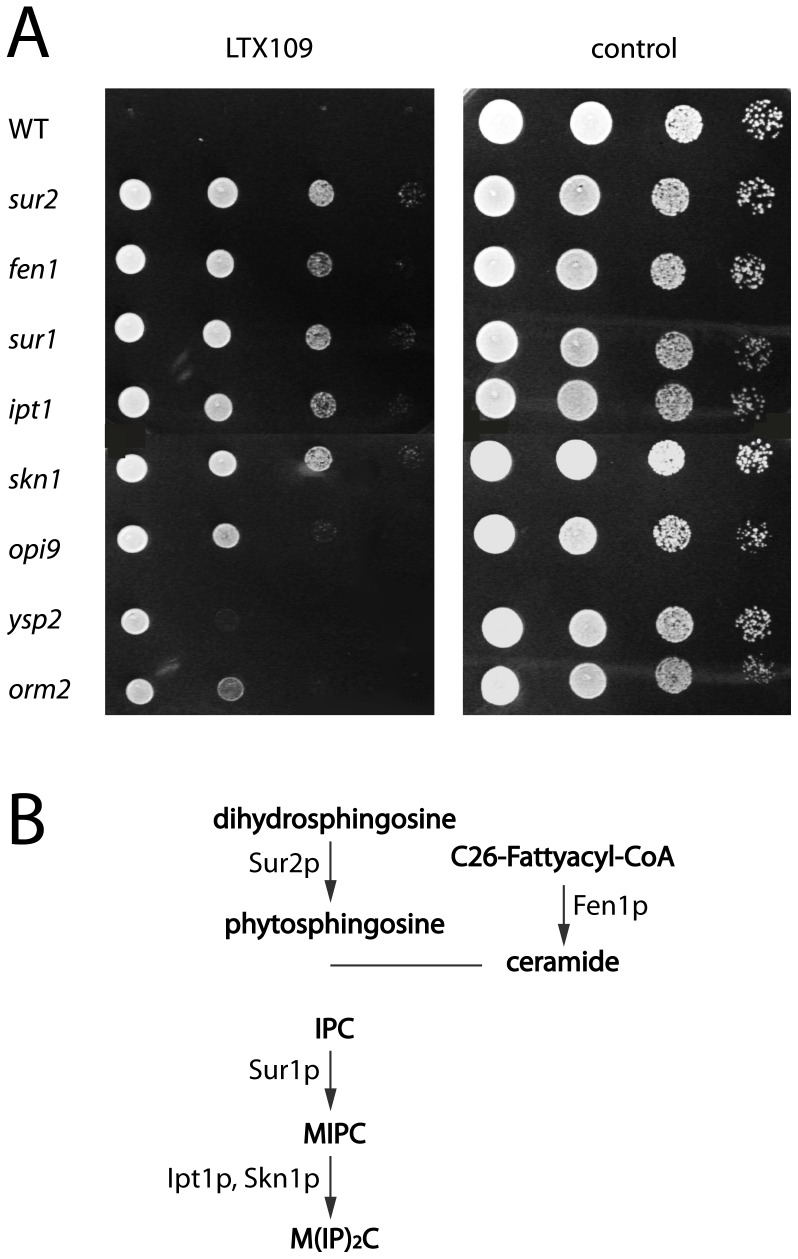
Mutants in sphingolipid biosynthesis are resistant to LTX109. (A) Spot test of wild type (WT) *S. cerevisiae* and eight deletion mutants identified by screening a deletion collection for LTX109 resistance. Exponentially growing yeast was resuspended in YPD to 10^7^ cells/ml and serially diluted 1∶10. Aliquots (6 µl) were spotted on solid YPD plates containing 70 µg/ml LTX109 (left panel), and without LTX109 (right panel). (B) *S. cerevisiae* sphingolipid biosynthetic pathway.

To gain further insight into the LTX109 mode of action, we screened a collection of haploid *S. cerevisiae* knockout mutants for LTX109 resistance. We isolated 17 mutants that were resistant to LTX109 at 10 times the MIC on YPD agar. Eight genes conferred LTX109 resistance when deleted (Table 1; Fig. 4A). Six of the identified genes (*SUR1*, *FEN1*, *SUR2*, *IPT1*, *SKN1*, *ORM2*) were involved in the biosynthesis of sphingolipids, which are a major plasma membrane component. Fen1p and Sur2p are involved in synthesis of ceramides, which are precursors for inositol phosphoceramide (IPC), the first complex sphingolipid in the synthesis pathway [39] (Fig 4B). Fen1p elongates long-chain fatty acids that are linked to a sphingoid base to form ceramides [40] and Sur2p hydroxylates dihydrosphingosine (DHS) to form phytosphingosine (PHS) [41], which is the most abundant sphingoid base in yeast ceramides [39]. Sur1p mannosylates IPC to form the intermediate sphingolipid mannose inositol phosphoceramide (MIPC) [42] and Skn1p and Ipt1p have similar functions in the biosynthesis of the terminal sphingolipid mannosyl di-inositol phosphorylceramide (M(IP)_2_C) [43]. Orm2p is a regulator of the sphingolipid biosynthesis that links the biosynthesis to the regulatory Target Of Rapamycin pathway [44]. Mutants that fail to activate Orm2p have reduced levels of sphingolipids as do *fen1, sur1, ipt1* and *skn1* mutants [40], [43], [45], [46], suggesting a role of sphinolipids in sensitivity to LTX109.

**Table 1:** *S. cerevisiae* genes that confer LTX109 resistance upon deletion.

Deleted gene and function	ORF	Gene product	n
**Sphingolipid biosynthesis**			
*SUR1*	YPL057C	Mannosylinositol phosphorylceramide (MIPC) synthase catalytic subunit	2
*SUR2*	YDR297W	Sphinganine C4-hydroxylase	8
*ORM2*	YLR350W	Sphingolipid homeostasis. Interacts with serine palmitoyl transferase (SPT)	1
*IPT1*	YDR072C	Inositolphosphotransferase, involved in synthesis of mannose-(inositol-P)2-ceramide (M(IP)2C)	1
*FEN1*	YCR034W	Involved in membrane-bound fatty acid elongation up to 24 C (ceramide precursor)	1
*SKN1*	YGR143W	Involved in the terminal M(IP)C →M(IP)2C process	2
**Apoptosis**			
*YSP2*	YDR326C	Mitochondrial protein in programmed cell death.	1
**Unknown function**			
*OPI9*	YLR338W	Dubious ORF unlikely to encode a protein. Partly overlaps *VRP1*	1

n, number of mutants identified.

One LTX109-resistant mutant was affected in the *YSP2* gene, which is involved in apoptosis, and another was affected in *OPI9*. *OPI9* has an unknown function but partly overlaps with *VRP1*, which encodes an actin-associated protein with a role in actin filament organization. The *opi9* mutant therefore also has a partial deletion of *VRP1*, so the LTX109-resistance phenotype could be caused by loss of Vrp1p activity. Resistance of each mutant was confirmed by spot-testing diluted yeast suspensions on YPD agar containing LTX109 (Fig. 4A). Five of the mutants affected in sphingolipid biosynthesis showed similar, high resistance towards LTX109 (*sur1*, *fen1*, *sur2*, *ipt1* and *skn1*).

### LTX109 efficiently kill S. cerevisiae growing as biofilm

**Figure 5:**
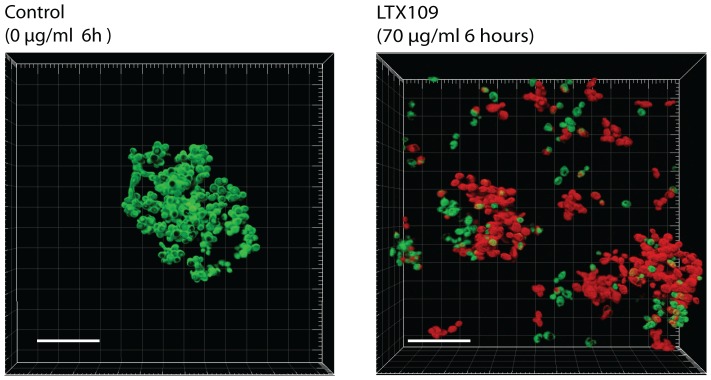
Activity of LTX109 against yeast biofilm. Confocal Laser Scanning Microscopy of *S. cerevisiae* (Σ1278*b*) biofilm. Cells were grown in Lab-Tek™ Chamber Slide™ System; Permanox - (NUNC, Denmark) in 1 ml synthetic complete medium After 12 hours, the cells were exposed to 0 µg/ml LTX109 (control) or 70 µg/ml LTX109 for another 5 hours. The biofilm cells were then stained with Syto9 (green) and propidium iodide (red) LIVE/DEAD stain before confocal laser scanning microscopy. Images are 3D reconstructions of biofilm made from 2 µm thick images in stacks of 20 individual images. CLSM was perform with a Zeiss LSM510 microscope using a 63x/0.95NA a water immersion lens. Life dead staining of biofilm treated with LTX109 was repeated in four independent experiments. White bar is 30 µm.

Because S288c is incompetent of biofilm growth [47], [48] we used the Σ1278*b* strain background to test the antifungal activity of LTX109 against *S. cerevisiae* biofilm. To visualize the antifungal properties of LTX109, we used CLSM in combination with Syto 9 DNA viability stain and propidium iodide that only penetrates damaged cell membranes. Intermediate (12 h) *S. cerevisiae* biofilm grown in batch culture slides were treated with 10 times MIC LTX109 for 5 hours before LIVE/DEAD staining and CLSM (Fig. 5). The LTX109 treatment killed the majority of the biofilm population as indicated by uptake and staining of dead cells with propidium iodide (Fig. 5), suggesting that LTX109 is also an efficient anti-biofilm agent in addition to its fungicidal activity against planktonic cells in exponential growth phase.

## Discussion

This study demonstrated the fungicidal activity of the peptidomimetic LTX109. Antimicrobial peptidomimetics are peptide-like compounds, of which most are bactericides [49]–[53]. LTX109 was previously shown to have bactericidal activity [27]. The arginine–tertbutyl tryptophan–arginine sequence of LTX109 makes it amphipathic, with two bulky side groups and two cationic side groups that are proposed to interact with negatively charged bacterial cell membranes [26].

We found similar killing kinetic for LTX109 and the membrane permeabilizing drug amphotericin B, suggesting that the two compounds could have a related mode of action. The rapid uptake of the fluorescent dye SYTOX Green, potassium and amino acid efflux from cells exposed to a high concentration of LTX109 suggest that this drug disturbs the plasma membrane by direct interaction with one or several components in the plasma membrane. Furthermore, inability of LTX109 treated cells to acidify their surrounding media support an effect on the cell membrane.

These results are similar to results with amphotericin B, which also causes yeast cells to inhibit glucose-induced acidification [32] and to release potassium as a consequence of general membrane disorganization [54], [55].

The high concentration of drug could have obscured other toxic effects of LTX109 on *S. cerevisiae*, so we cannot exclude that LTX109 has other effects in addition to membrane disruption as previously discussed for peptide drugs [56].

To gain further insight into the mode of function of LTX109, we screened for resistant mutants. Six of eight resistance mutants were affected in sphingolipid biosynthesis, and five of these showed similar, high resistance towards LTX109 (Fig. 4). *fen1, sur1, ipt1* and *skn1* mutants all have reduced amount of sphingolipids [40], [43], [45], [46] as do mutants that reduce Orm2p activity [44], suggesting an essential role of complex sphingolipids in sensitivity to LTX109. Lack of Sur2p lead to decreased sphinganine hydroxylation, but does not prevent formation of MIPC [57]. Furthermore, the *fen1* mutant produce reduced amount of sphingolipids containing the C26 acyl group [40]. The resistance phenotype of the *sur2* and *fen1* mutants therefore suggested that it is not only the quantity, but also the structural modifications that occur during sphingolipid synthesis that is required for optimal LTX109 activity. The terminal steps of sphingolipid biosynthesis in yeasts are MIPC and M(IP)_2_C. The fact that these lipids are reduced in the resistant mutants suggests that MIPC and M(IP)_2_C are essential for the fungicidal activity of LTX109, either by direct interaction with LTX109 or by interaction with another membrane components that is the target for LTX109. It does however seem less likely that a component other than sphingolipids is the target for LTX109 for two reasons, (i) mutants depleted of the target would be expected to appear in the screen for mutants resistant to LTX109. (ii) Alternatively, the target could depend on sphingolipids for optimal activity, be essential for growth and thus not appear in the screen, but then *fen1, sur1, ipt1*, *skn1* and *orm2* mutants would be expected to have reduced growth rates which they do not (Fig. 4).

Sphingolipids are located primarily in the plasma membrane [58] and are often clustered together with ergosterol in lipid rafts [59]. Sphingolipids are not only a structural component of the cell membrane, but serve vital functions in the heat-shock response, cell cycle arrest, signaling pathways, endocytosis and protein trafficking [60], [61]. Fungal sphingolipids are highly similar to each other [62], [63], and the biosynthesis of complex fungal sphingolipids is distinctly different from mammals [64]. This makes the fungal sphingolipids attractive antifungal drug targets and several natural compounds with anti-IPC synthase activity have been identified [65]–[67].

The terminal M(IP)_2_C is the major sphingolipid in the fungal plasma membrane [46] and has previously been suggested as a target for the plant defensin *Dahlia merckii* antimicrobial peptide 1 (DmAMP1) [43], [68], [69]. DmAMP1 is a 50 amino acid amtimicrobial peptide that leads to nonselective passage of potassium, calcium [70] and SYTOX Green [33]. Hence, DmAMP1 and LTX109 could have similar modes of action, although DmAMP1 does not contain the Arg-Trp-Arg sequence that serves as basis for LTX109.

Amphotericin B is currently the last in line treatment option for severe fungal infections [71]. Alternative drug candidates might therefore be developed for treatment in cases where use of amphotericin B becomes limited due to resistance. Biofilm formation on medical devices is a major nosocomial problem and causes multidrug resistance [13]. Only a few of the current systemic antifungals have activity against yeast biofilms [72], [73], but often it requires removal of the implant for effective treatment [74]. Peptide antibiotics including LTX109 analogues have been shown to be efficient drugs to kill bacterial biofilm cells [25], [50]. This study shows for the first time a peptidomimetic with activity against yeast biofilm. This observation suggests antifungal peptidomimetics with rapid killing kinetics and membrane permeabilizing activities are attractive drugs for yeast biofilm treatment.

In conclusion, we have shown the efficient fungicidal properties of a synthetic peptidomimetic, LTX109, that killed the yeast *S. cerevisiae* with fast killing kinetics and complete eradication of viable cells in exponential growth phase. We found that yeast cells treated with a high concentration of LTX109 became permeable to free amino acids, potassium and SYTOX Green and prevented proton extrusion in response to a pulse of glucose. Fungal susceptibility to LTX109 depended on biosynthesis of sphingolipids. The sphingolipids M(IP)_2_C and its precursor MIPC are found in fungal, but not human membranes, making LTX109 and derivatives attractive drug candidates for fungal infection treatment as alternatives to amphotericin B.
